# The association of cervicovaginal Langerhans cells with clearance of human papillomavirus

**DOI:** 10.3389/fimmu.2022.918190

**Published:** 2022-10-12

**Authors:** Wenkui Dai, Liming Gui, Hui Du, Shuaicheng Li, Ruifang Wu

**Affiliations:** ^1^ Department of Obstetrics and Gynecology, Peking University Shenzhen Hospital, Shenzhen, China; ^2^ Institute of Obstetrics and Gynecology, Shenzhen Peking University-Hong Kong University of Science and Technology Medical Center (PKU-HKUST) Medical Center, Shenzhen, China; ^3^ Shenzhen Key Laboratory on Technology for Early Diagnosis of Major Gynecologic Diseases, Shenzhen, China; ^4^ Department of Biomedical Engineering, City University of Hong Kong, Hong Kong, Hong Kong SAR, China

**Keywords:** human papillomavirus clearance, Langerhans cells, cervicovaginal microbiota, cellular immunity, human papillomavirus infection

## Abstract

Human papillomavirus (HPV) clearance is important in eliminating cervical cancer which contributes to high morbidity and mortality in women. Nevertheless, it remains largely unknown about key players in clearing pre-existing HPV infections. HPV antigens can be detected by the most important cervical antigen-presenting cells (Langerhans cells, LCs), of which the activities can be affected by cervicovaginal microbiota. In this review, we first introduce persistent HPV infections and then describe HPV-suppressed LCs activities, including but not limited to antigen uptake and presentation. Given specific transcriptional profiling of LCs in cervical epithelium, we also discuss the impact of cervicovaginal microbiota on LCs activation as well as the promise of exploring key microbial players in activating LCs and HPV-specific cellular immunity.

## Introduction

Persistent high-risk human papillomavirus (hrHPV) infections causes the highest risk of invasive cervical cancer (ICC), one of the most common cancers threatening women’s health worldwide (1). Prophylactic HPV vaccines can not cover all HPV subtypes in ICC cases and thus provide limited benefits to eliminate pre-existing HPV infections (2, 3) which affect large populations in developing countries. Though screening is effective in preventing the progression of HPV infection and cervical dysplasia to ICC, it takes a long time to make it widely utilized, especially in low-income countries due to high financial and human resource burden. Clearance of HPV infection is encouraging alternative to eliminate ICC. Our and other prospective observational studies indicated that 85%, 50% and 5-10% of HPV infections could be cleared spontaneously in cervical intraepithelial neoplasia (CIN) 1, 2 and 3 respectively (4–6). Nevertheless, mechanisms of natural HPV clearance were largely unknown.

Langerhans cells in cervicovaginal mucosa represented key players in HPV antigen presentation and cellular immunity activation (7, 8). Emerging studies found LCs number or maturation levels reduced after HPV infections, and implicated the association of LCs with HPV clearance as well as CIN regression (9–14). *In vitro* experiments also indicated that LCs activation primed T cells and caused HPV-specific cellular immunity (15–18). Recent studies indicated unique transcriptional profiling of LCs in cervicovaginal microenvironments as compared to the skin and blood (19, 20), which may be caused by the impact of *Lactobacillus* and *Candida* on LCs immune functions (21, 22).

This review discusses how cervicovaginal HPV infections impair LCs immune functions, and what factors in cervicovaginal microenvironment hold the potential to activate LCs promoting HPV clearance, all of which will provide extensive insights into ICC prevention.

## Carcinogenesis by persistent HPV infections

**Figure 1:**
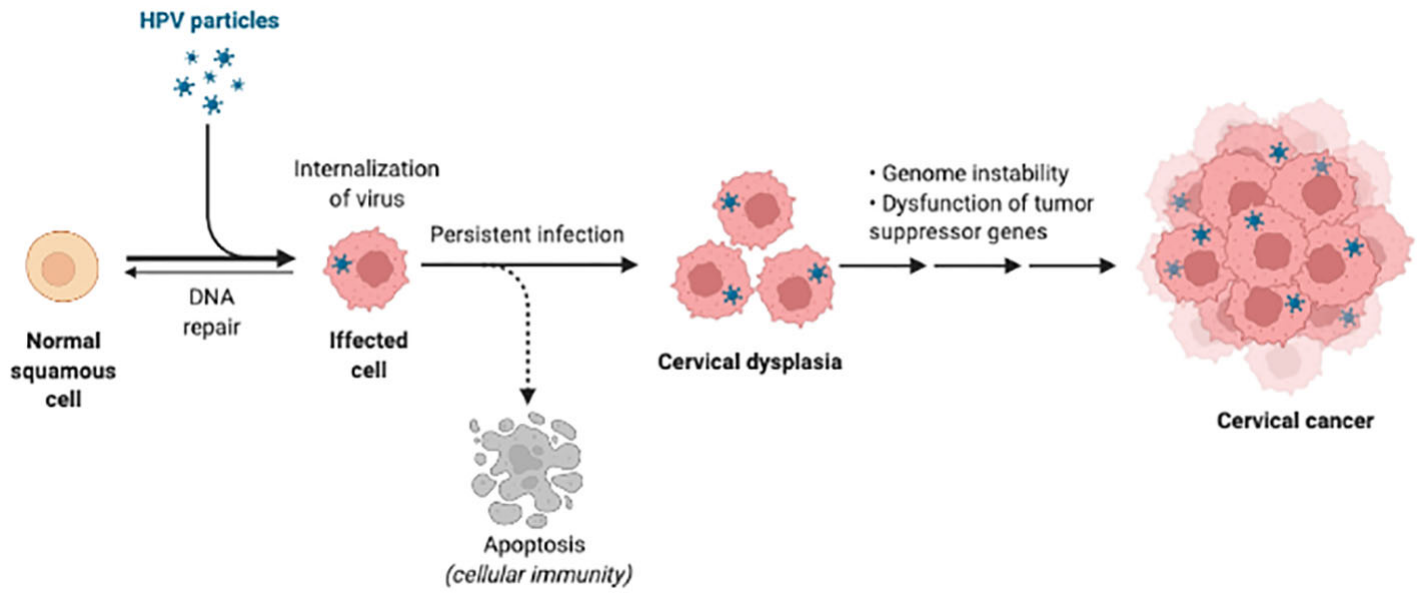
HPV infection and carcinogenesis. The HPV is internalized into the squamous cells first and persistent infection can cause DNA integration. Then infected cells are in dysfunction and develop to cancer. Created in BioRender.com.

HPV infections require access of viral DNA, capsid protein L1 and L2 to the basal lamina binding to heparin sulfate proteoglycan on basal keratinocytes (23, 24). Once internalized, virions undergo endosomal transport and uncoating, with L2 protein-DNA complex (episome) ensuring correct nuclear entry of viral genomes while L1 protein retained in the endosome being degraded (25, 26). Before entry to the nucleus, E2 protein inhibits the expression of E6/E7 proteins which directly relate to the increasing severity of neoplasia by driving cell proliferation as well as inducing immune system dysfunction (27–29). If HPV episomes escape from host immune clearance, accumulation of E6/E7-induced genetic errors will eventually promote integration of viral episomes into the host cell chromosome (30–32) (**Figure 1**), together with overexpression of E6 and E7 led by dysregulation of E2 protein. Life cycle of HPV during persistent infections completes through the expression of minor coat protein L2 and major coat protein L1, as well as virus maturation in dying keratinocytes.

Immune evasion plays key role in the above-mentioned life cycle of HPV. Keratinocytes function as immune sentinels *via* expressing pathogen recognition receptors (PRRs) to recognize pathogen-associated molecular patterns of HPV. Nevertheless, viral infection can suppress activities of PRRs such as toll-like receptors(TLRs) (33). For instance, E7 oncoprotein repressed TLR9 transcription by recruiting histone modifying enzyme EZH2 to the TLR9 promoter region (34). In consistence, E7 downregulates TLR9 expression through recruiting histone deacetylase HDAC1 and histone demethylase JARID1B to the regulatory region of the TLR9 promoter (35). Upon viral recognition, PRRs transduce intracellular signals to initiate the production of proinflammatory cytokines such as interferon(IFN)α and IFNβ. However, E6 and E7 proteins can block PRRs signal transduction cascades through two pathways: binding to interferon regulatory transcription factor (IRF) to inhibit its transcriptional activity and upregulating the deubiquitinating enzyme Ubiquitin C-Terminal Hydrolase L1 (UCHL1) to prevent TNF-receptor-associated factor 3 (TRAF3) activation (36–38).

The downregulation of NF-κB pathway is another critical strategy applied by HPV oncoproteins for immune evasion. NF-κB plays a key role in immune surveillance through promoting the expression of genes involved in antigen presentation and cytokine production (39). There is evidence supporting HPV16 E6 and E7 can inhibit NF-κB activity in keratinocytes which were cultured from the human cervical transformation zone (40). Moreover, high-risk HPV-infected keratinocytes upregulate UCHL1 to prevent the nuclear translocation of NF-κB (38). E6 and E7 can also bind to the coactivator of NF-κB [P300/CBP-associated factor (PCAF)] and then downregulate the NF-κB signaling pathway (41, 42).

Besides to impaired immune alarm functions of keratinocytes, above-mentioned inhibition of keratinocyte activities also modulates immune network to further facilitate persistent infection. For example, E5 protein reduces the expression of the major histocompatibility complex (MHC) I and CD1d to the cell surface, and then prevents cytotoxic T lymphocytes recognition of HPV antigens (43–45). In addition, E7 protein can interact with MHC I promoter, leading to repression of MHC I, LMP2, and TAP1 gene (46–48). Furthermore, HPV oncoproteins are capable of inhibiting the migration of antigen-presenting cells (APCs) to infected sites and repressing the APCs activities (49–53), further facilitating persistent infection.

## Suppressed LCs activities caused by cervical HPV infections

Since LCs are the only APCs with which HPV will come into contact during HPV infections and initiate cell-mediated immune responses against HPV (7, 8, 19, 20), LCs are the most important and primary antigen-presenting cells (APCs) in the cervical epithelium. Suppression of LCs activities facilitated an immunosuppressive microenvironment that is permissive for HPV persistence (51–53), which was documented by reduced LCs number and maturation levels in cervical epithelium under HPV infections and squamous intraepithelial lesion (9–12).

### Recruiting and remaining LCs in epidermis

**Figure 2:**
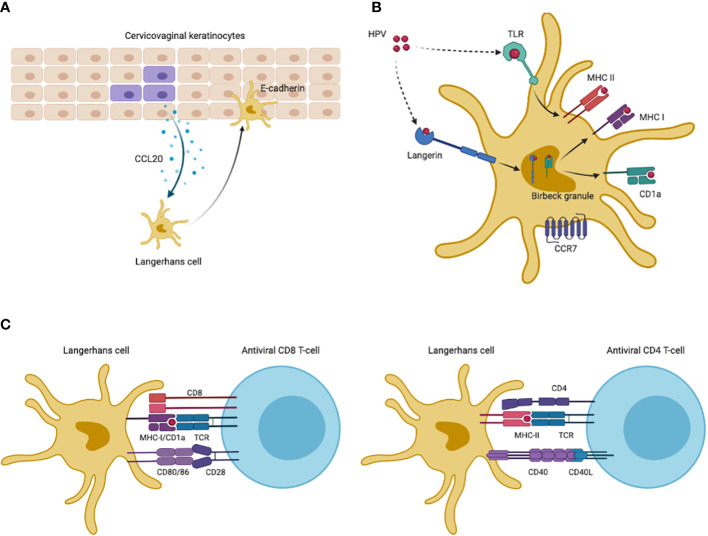
HPV antigen uptake and presentation of LCs. **(A)** Infected keratinocytes increase expression of CCL20 to attract Langerhans cells, which then migrate to inflammatory epidermis and adhere to keratinocytes *via* LCs-derived E-cadherin. **(B)** LCs maintained in inflammatory site capture HPV antigens *via* TLRs and langerin. Antigen uptake by TLRs are displayed on cell-membrane MHC-II molecules for presentation. Langerin-captured antigens are processed in intracellular Birbeck granules, being loaded on CD1a molecules in Birbeck granules or displayed in MHC-I molecules. CD1a molecules then cycle back to cell surfaces to present antigens. After antigen uptake and processing, LCs are migratory to lymph nodes *via* CCR7 which are receptors for T cell-derived CCL21. **(C)** Co-stimulatory molecules promote antigen presentation to CD4+ and CD8+ T cells. This Figureure was created in BioRender.com.

Monocytes should be differentiated into competent and immature Langerhans cells which can detect pathogenic antigens. Nonetheless, HPV infection can block this differentiation and then inhibit the production of LCs (15). Immature LCs maintain the immune surveillance of the epithelium by presenting antigens and stimulating T lymphocytes following antigen uptake. After viral infections, immature LCs are recruited to the epidermis *via* chemokine (C-C-motif) receptor 6 (CCR6), which is the receptor for chemokine (c-c-motif) ligand 20 (CCL20) increasingly expressed by infected cervical keratinocytes (54, 55). Then LCs adhere to infected keratinocytes *via* LCs-expressed E-cadherin, as shown in **Figure 2A**. Nevertheless, HPV was reported to interfere with keratinocyte-derived CCL20 expression and HPV E6/E7 proteins promoted downregulation of CCL20, inhibiting LCs migration to the epidermis of inflammation (49, 50). Reduced E-cadherin levels caused by E7 protein also suggested the blockade of LCs adhesion with infected keratinocytes (56, 57), negatively affecting antigen uptake in epidermis after HPV infections. Moreover, the LC-keratinocyte crosstalk was dysregulated including decreased expression of interleukin-34 and depletion of immune-stimulatory LCs (58).

### Capturing and processing antigens by LCs

When remained in cervical epidermis, immature LCs can capture HPV antigens *via* C-type lectin langerin (CD207) and toll-like receptors (TLRs) (59), as shown in **Figure 2B**. Cell-membrane langerin functions as endocytic receptor and internalizes antigens to LCs-specific intracellular Birbeck granules where antigens are degraded or delivered to antigen-presenting CD1a and major histocompatibility complex I (MHC-I) (60–64). Nevertheless, prior reports suggested that HPV significantly impaired langerin-CD1a antigen processing (10, 12). Besides to langerin, LCs also capture HPV antigens *via* TLRs which are common pathogen-recognition receptors (PRRs) on cell surface (59). However, LCs isolated from cervical tumors had decreased TLRs expression, such as TLR7/8/9, and were functionally anergic to TLR ligands, while a number of studies found that selective TLR agonists could promote the maturation of LCs (65–69). Additionally, HPV oncoproteins can activate the phosphoinositide 3-kinase (PI3-K) pathway to inhibit immune responses to HPV infection (16).

### Migration of mature LCs to lymph node

LCs downregulate their endocytic and antigen processing capacity after binding pathogens, and then become mature as well as migratory (70). To present captured antigens, LCs should migrate to the lymph nodes *via* CCR7 which is only expressed by mature LCs and functions as receptor for T cell-derived chemokine CCL21 (55, 71), as shown in **Figure 2B**. Though there lacked reports on CCR7 levels of LCs after HPV infections, cervical cancer cells suppressed the induction of CCR7 in phenotypically mature dendritic cells (DCs), while specific co-stimulatory molecules upregulated CCR7 expression on LCs surface (65, 68, 72, 73).

### Presenting antigens *via* LCs to activate cell-mediated immunity

CD1a, which is a lipid-presenting molecule abundantly expressed on LCs, can bind antigens in Birbeck granules and recycle to the cell surface presenting lipid/glycolipid antigens to CD1a-restricted T cells (63). HPV infections reduced the density of CD1a+ LCs in cervical epithelium, and CD1a+ LCs number correlated positively with prognosis of HPV-infected cases (10, 12, 13). Cell-membrane MHC-I molecules cross-present pathogen-derived peptides which can be recognized by cytotoxic lymphocytes, while MHC-II molecules are mainly expressed on APCs surfaces presenting antigens to helper T cells. HPV E7 protein was reported to directly block the MHC-I heavy chain promoter and E5 protein retained human leukocyte antigen A/B (HLA-A/B) (44, 46, 74), which may explained the partial loss of MHC-I in cervical cancer (75). MHC-II levels on LCs surface also reduced in cervical biopsy collected from CIN patients (11), while TLR agonists activating LCs immune functions or bacterial vector vaccines could up-regulate expression of MHC molecules and elicit CD4+/CD8+ CMI (65, 68, 76, 77).

Unlike typical DCs, co-stimulatory molecules CD80/86 and CD40 on LCs, belonging to tumor necrosis factor (TNF) receptor family, are needed in presenting antigens to T cells and inducing CMI (78–81) (**Figure 2C**). CD80/86 binds T cell-derived CD28 to prevent the induction of T cell anergy, while the CD80/86 ligand (cytotoxic T lymphocyte associate protein-4, CTLA-4) plays an inhibitory role by inducing T cell anergy. CD40 is another type of TNF receptors on LCs and functions by binding T cell membrane CD40L to prevent anergic status of T cells. *In vitro* experiments found higher levels of CD80/86 and CD40 in monocytes-derived LCs exposed to HPV VLPs after introduction of TLR agonists (65, 68).

## Modulation of LCs activities and clearance of HPV infections in cervicovaginal microenvironment

Given the key roles of mucosal LCs in presenting HPV antigens and inducing CMI in cervical microenvironment, activation of suppressed LCs holds the potential to promote HPV clearance, partially supported by prior reports suggesting that LCs number was a strong and independent prognostic factor for HPV-infected cases in cervical, head and neck, lung carcinoma (13, 14, 82). Dorotheíe Duluc et al. identified cervical mucosa-specific LCs transcriptional fingerprints compared to skin and blood (19, 20), suggesting the impact of cervicovaginal microenvironment on LCs activities.

### The complexity of cervicovaginal microbiota

A number of studies found predominance of one or few *Lactobacillus* species in CVM in healthy lower reproductive tract, like *Lactobacillus crispatus* (community-state type I, CST I), *Lactobacillus gasseri* (CST II), *Lactobacillus iners* (CST III) and *Lactobacillus jensenii* (CST V) () (83–87). In contrast, depletion of *Lactobacillus* (CST IV) was frequently found in women with genital infections (88–95). Microbe-microbe and -host interactions determined the complexity of CVM. Vaginal *Lactobacillus* spp. can produce lactic acid *via* glycogen fermentation, maintaining acidic environment to inhibit the colonization of pathogens such as *Chlamydia trachomatis*, *Neisseria gonorrhoeae* and *Gardnerella vaginalis*. In addition, *Lactobacillus*-derived bacteriocins exhibited inhibitory effects on common pathogenic bacteria and certain fungi, like *G. vaginalis* and *Candida* (96–98). Biosurfactants excretion was also applied by *Lactobacillus* to alter surface tension and bacterial adhesion, thus then preventing overgrowth of pathogenic anaerobes, especially *G. vaginali*s (99–102). Additionally, CVM can modulate a finely-tuned immune response balancing reproductive tolerance with protection against genital infections (83, 103, 104).

### The association of CVM with HPV infections and clearance

**Figure 3:**
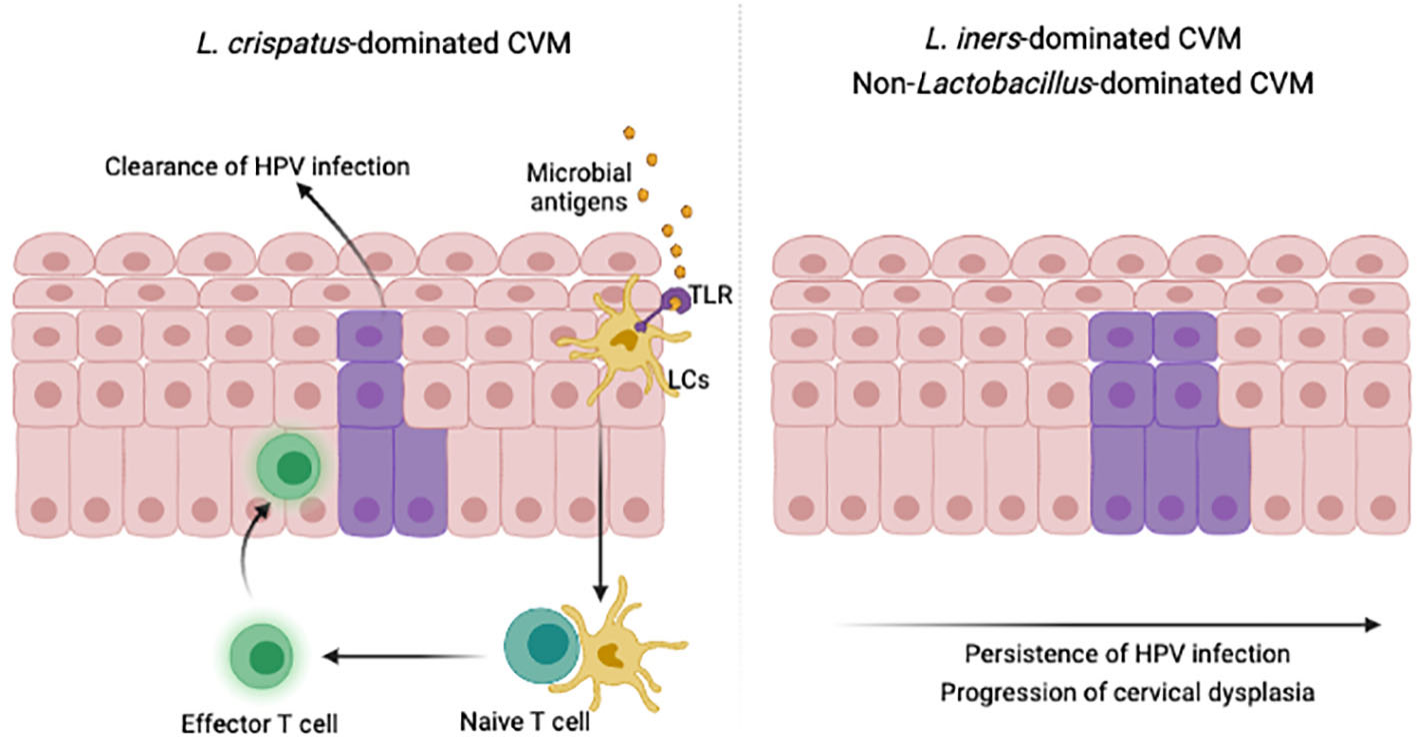
CVM modulate immunity to promote HPV clearance. Microbial key players in specific cervicovaginal environment activate suppressed LCs, migrating to lymph nodes, presenting antigens and attracting effector T cells to infected epidermis. This illustration was created in BioRender.com.

Imbalanced CVM was widely identified in women with persistent HPV infections and cervical intraepithelial neoplasia (CIN), including decreased bacterial diversity, depletion of Lactobacillus and accumulation of *Gardnerella* and *Sneathia* (88–93). Further analysis found significant differences of CVM and cervical immune microenvironment between HPV- or CIN-negative women and HPV-positive women with CIN or cervical cancer (105–108). For example, inhibitory immune checkpoint protein PD-L1 and LAG-3 were in negative correlation with *Lactobacillus* levels, whereas TLR2 correlated positively with *Lactobacillus* abundance. In contrast, PD-L1 and LAG-3 positively correlated with pathogen *Gardnerella*, *Sneathia*, *Atopobium* and *Prevotella*. Additionally, *L. jensenii* and *L. crispatus* were in negative relationship with PD-L1, and *L. gasseri* was negatively associated with LAG-3. A 12-month observational study also demonstrated the critical role of cervicovaginal bacteria in modulation of cervicovaginal immune responses and the host susceptibility to HIV (103). Additional studies showed that *L. cripatus*-dominated CVM provided higher protection against pathogenic infections, compared to *L. iners*-dominated and non-*Lactobacillus*-dominated CVM (109–112) (**Figure 3**). Longitudinal studies further suggested high proportion of HPV clearance or CIN regression for HPV-positive populations with dominant *L. crispatus* in CVM (111, 112). Besides to bacterial components in CVM, vaginal fungi were associated with persistence of HPV infections (112), though a retrospective study involving 100,605 women found that *Candida* was not in significant association with the risk of CIN (113).

### Modulating LCs activities *via* cervicovaginal microbial components

Though the potential role of CVM in modulating immune responses is largely unknown, co-culturing of monocytes-induced LCs and vaginal *Lactobacillus* indicated that peptidoglycans (PGN) in cell wall of specific *L. crispatus* strain increased the levels of LCs surface marker (langerin) which is pivotal in antigen capture (21). Further analysis demonstrated that PGN induced higher levels of langerin *via* elevated expression of recognized receptors of *L. crispatus*, such as TLR2 and TLR6 (21). Other studies also suggested that lipid polysaccharides (LPS), common bacterial product and potent TLR4 agonist activated LCs being assessed by upregulated MHC-II and CD80/86 molecules (72).

Given effective role of TLR agonists in therapeutic vaccines against HPV infections (114–119), emerging reports suggested the promise of selective microbial components as “natural” adjuvant in therapeutic HPV vaccines (111–125), partly due to their potential in enhancing LCs functions *via* activating TLRs through wide range of pathogen-associated molecular pattern molecules and damage-associated molecular pattern molecules. For instance, mouse model indicated that *Lactobacillus lactis* and *Lactobacillus casei* immobilizing HPV E7 peptides were effective in inducing HPV-specific CMI (124, 125). Candin, produced by common vaginal fungi *Candida*, was also applied as effective adjuvant in therapeutic vaccine against HPV infections, inducing wart resolution and elevated T-helper type 1 cells which promoted viral clearance (111, 112).

## Concluding remarks and future perspectives

Cervicovaginal LCs play an important role in recognizing HPV antigens and activating HPV-specific CMI, and prior reports found the association of natural clearance of HPV infections with increased LCs activities (13, 14, 82). Though some LCs activators, like TLR agonists and products of vaginal microbes can activate LCs (21, 22, 65, 68), associated adjuvants were not always effective in promoting HPV clearance and CIN regression (114–116, 118–125). This may be explained by inter-individual CVM differences which can impact LCs functions as well as natural clearance of pre-existing HPC infections. Nevertheless, it remains unclear for the mechanisms how CVM modulate LCs activities to promote HPV clearance. Though *in vitro* experiments indicated the positive impact of specific vaginal *Lactobacillus* strains on LCs activities (21), functional redundancy for microbiota (126–128) and *Lactobacillus* strain-specific functions (129–133) necessitated the exploration of “functionally key microbial products” in modulating LCs activities.

To determine the promise of activating cervicovaginal LCs as novel therapies to clear HPV infections, the following research strategies are recommended. Firstly, prospective cohort study on HPV-positive women without CIN or with low-grade CIN are conducted to analyze the association of CVM-host interactions with clinical outcomes of HPV infections in cervicovaginal microenvironment. Secondly, multi-omics technologies, such as meta-transcriptomics, should be applied to explore the candidates of microbial products which can increase the level of infected keratinocyte-derived CCL20, attracting migration of LCs to HPV-infected site. Additionally, we also need to analyze candidate microbial products and associated gene sequences which hold the potential to activate LCs as TLR agonists or co-stimulatory molecules. Thirdly, *in vivo* cellular and animal models should be applied to assess the role of aforementioned microbial products in modulating LCs activities and clearing HPV, including W12 cell lines which were isolated from women with HPV16 infection and low-grade CIN as well as dog, rabbit and mouse models with relative papillomavirus (134). Lastly, sub-clinical and even clinical trial will be applied to assess the efficacy of above-mentioned microbial products in activating LCs and promoting HPV clearance.

Though activating cervicovaginal LCs is promising in clearing HPV infections, more solid evidence will be needed to explore the association of LCs activities with HPV clearance. For instance, the NF-κB signaling pathway that can regulate the expression of CCL20 and a variety of cytokines was inhibited in HPV-associated pre-cancerous CIN but activated significantly in cervical cancer (38–42, 135), which can be explained by dramatically changed immune microenvironment for cervical cancer (39, 135–138). Given ever-updating findings on HPV-host interaction (138–140), immune microenvironments such as lymph nodes and LCs-related T cell responses should be considered when exploring the role of CVM-LCs interactions in HPV clearance.

## Author contributions

WD, LG and RW made substantial contributions to the design and writing of this manuscript. HD and SL contributed to the discussion and conception of the work. All authors contributed to the article and approved the submitted version.

## Funding

This work was supported by Shenzhen High-level Hospital Construction Fund (YBH2019-260), Shenzhen Key Medical Discipline Construction Fund (No.SZXK027) and Sanming Project of Medicine in Shenzhen (No.SZSM202011016) and Scientific Research Foundation of PEKING UNIVERSITY SHENZHEN HOSPITAL (No. KYQD2021075).

## Conflict of interest

The authors declare that the research was conducted in the absence of any commercial or financial relationships that could be construed as a potential conflict of interest.

## Publisher’s note

All claims expressed in this article are solely those of the authors and do not necessarily represent those of their affiliated organizations, or those of the publisher, the editors and the reviewers. Any product that may be evaluated in this article, or claim that may be made by its manufacturer, is not guaranteed or endorsed by the publisher.
